# Near-Infrared Inorganic Nanomaterials for Precise Diagnosis and Therapy

**DOI:** 10.3389/fbioe.2021.768927

**Published:** 2021-10-26

**Authors:** Wenling Li, Guilong Zhang, Lu Liu

**Affiliations:** Medicine and Pharmacy Research Center, School of Pharmacy, Binzhou Medical University, Yantai, China

**Keywords:** NIR imaging, inorganic nanomaterials, drug delivery, *in vivo*, precise diagnosis

## Abstract

Traditional wavelengths (400–700 nm) have made tremendous inroads in vivo fluorescence imaging. However, the ability of visible light photon penetration hampered the bio-applications. With reduced photon scattering, minimal tissue absorption and negligible autofluorescence properties, near-infrared light (NIR 700–1700 nm) demonstrates better resolution, high signal-to-background ratios, and deep tissue penetration capability, which will be of great significance for in-vivo determination in deep tissue. In this review, we summarized the latest novel NIR inorganic nanomaterials and the emission mechanism including single-walled carbon nanotubes, rare-earth nanoparticles, quantum dots, metal nanomaterials. Subsequently, the recent progress of precise noninvasive diagnosis in biomedicine and cancer therapy utilizing near-infrared inorganic nanomaterials are discussed. In addition, this review will highlight the concerns, challenges and future directions of near-infrared light utilization.

## Introduction

Biomedical imaging serves as an intermediary between physiopathology relevant information and clinical bio-applications. With the development of various clinical imaging modalities, series of fundamental research have obtained significant progress. Current imaging techniques including computed tomography (CT), positron emission tomography (PET), magnetic resonance imaging (MRI), ultrasound (US), photoacoustic imaging (PA), and single-photon emission tomography (SPECT), have been widely applied in disease diagnosis and treatment (Kim et al., 2018). However, some of these methods are not suitable for precise diagnosis. Exploring imaging modalities with non-invasiveness, better temporal-spatial resolution and higher sensitivity are the developing trend for precision medicine.

Fluorescence imaging has attracted growing interest for biosensing and bioimaging application especially in the near-infrared window (NIR, 700–1700 nm) (Reineck and Gibson, 2017). In the process of fluorescence imaging in most mammalian tissues, the photons penetration mainly relies on the light scattering, absorption and tissue autofluorescence, which will further restrict biomedical application (Carr et al., 2018). Compared with the visible spectrum (400–700 nm) extensively employed for bioimaging the broadly defined NIR region can provide deeper tissue optical imaging with improved signal-to-background ratio (Hong et al., 2017; Cao et al., 2019; Chen G. et al., 2020). Therefore, the increasing demand for fluorescence with high spatiotemporal resolution imaging in deep tissue calls for great progress in fundamental science both in better imaging instrumentation and new fluorescent agents.

Recent years, the emission wavelength of fluorescent agents gradually shift to the NIR region for fluorescent imaging. Organic fluorophores have become increasingly attractive for bioimaging and biosensing. For example, FDA approved NIR dye indocyanine green (ICG) has been widely used for clinical practices (Hu et al., 2016). However, the poor stability and solubility, low quantum yield of ICG remains an issue (Gil et al., 2021). Inorganic NIR emitters mainly including single-walled carbon nanotubes, rare-earth nanoparticles, quantum dots, metal nanomaterials are extremely important in NIR fluorescent imaging (Zhao et al., 2019). There is an ongoing effort toward the functional modification of NIR inorganic fluorophores as they can consist of different functional components in a single unit. Thus, multifunctional inorganic nanomaterials are used in bioimaging, therapy, drug delivery and precise diagnosis, etc.,.

This review aims to summarize recent progress of the NIR inorganic nanomaterials. We describe recent advances NIR contrast agents including single-walled carbon nanotubes, rare-earth nanoparticles, quantum dots, metal nanomaterials, including their emission mechanism. Then an overview of the use of these NIR inorganic nanomaterials in various bioimaging and biosensing applications are given. Finally, we conclude the overall concerns, challenges and future directions in the design of NIR fluorescent inorganic nanoprobes.

## Emission Mechanism of Near-Infrared Inorganic Nanomaterials

### Single-Walled Carbon Nanotubes

Single-walled carbon nanotubes (SWCNTs) quasi-one dimensional carbon based nanomaterials are considered as the rolling tube of a single carbon graphite sheet in a honeycomb lattice. SWCNTs were discovered by sumio lijima during the synthesis of the C60 in 1991 (Iijima, 1991). Benefit from its unique structure and excellent electrical, mechanics, carbon nanotubes have been rapidly used in physics, chemistry and biology. Moreover, SWCNTs have attractive intrinsic near-infrared emission (700–1700 nm) within the tissue transparency window.

**FIGURE 1:**
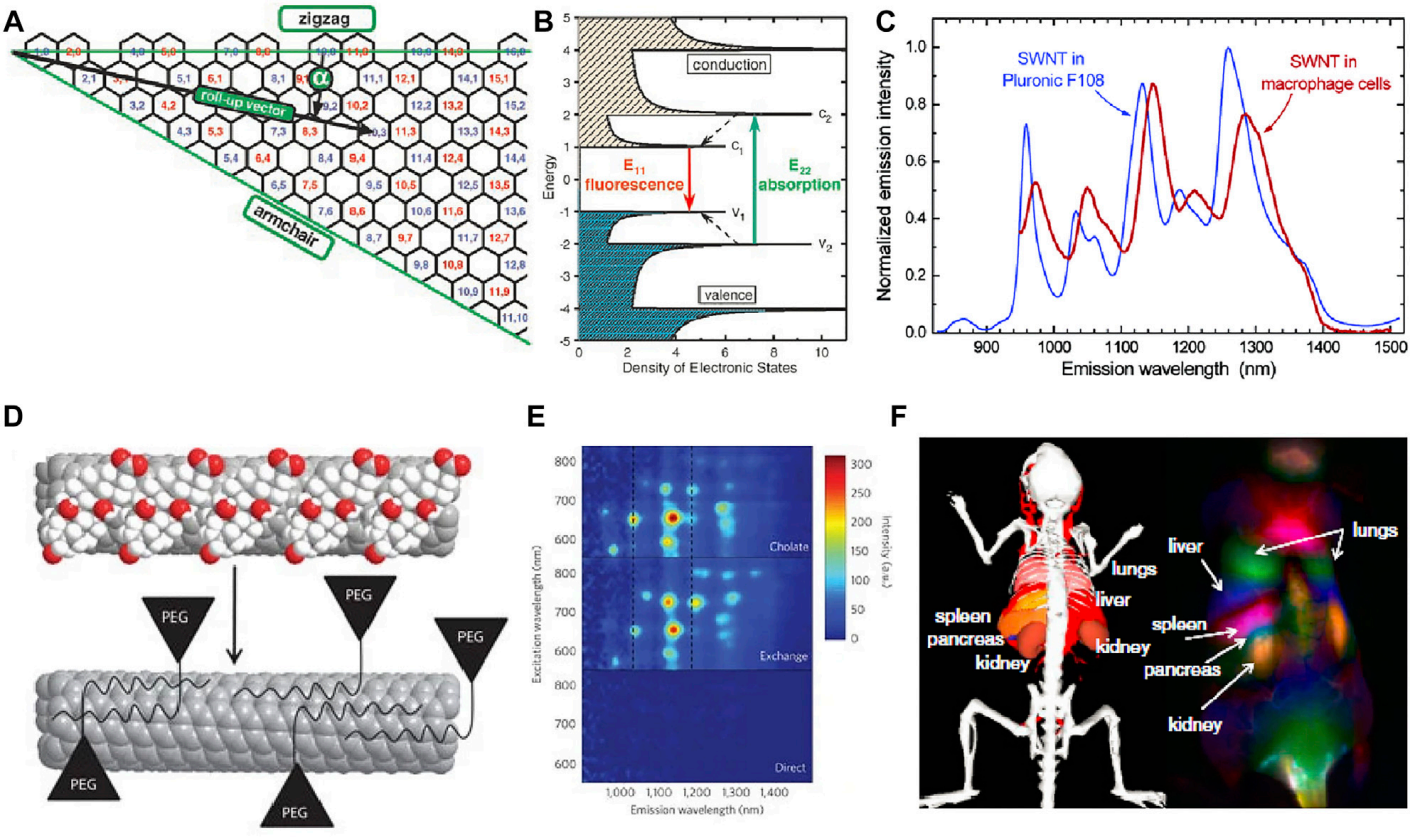
**(A)** Schematic of the graphene sheet segment showing roll-up vector. **(B)** Schematic density of electronic states for SWCNTs with van Hove singularities (Bachilo et al., 2002) Reproduced with permission from Weisman et al. (2002). **(C)** SWCNT emission spectra in an aqueous Pluronic F108 suspension and in macrophage cells (Cherukuri et al., 2004). Reproduced with permission from Weisman et al. (2004). **(D)** Schematic of the phospholipid-polyethylene glycol modified biocompatible nanotubes. **(E)** Photoluminescence versus excitation spectra (Diao et al., 2012). Reproduced with permission from Dai et al. (2012). **(F)** Dynamic contrast-enhanced imaging with SWNCTs through PCA (Welsher et al., 2011). Reproduced with permission from Dai et al. (2011).

SWCNTs have such special optical performance due to the presence of the Van Hove transitions across bandgap. M. J. O’Connell et al. discovered bandgap fluorescence of SWCNTs isolated inside surfactant micelles (Chen et al., 1998). The NIR emission is related to the energy band structure. The electron is pumped to excited states when light is initially absorbed. Subsequently, the emission can be measured during the electron relaxation from excited states to the lower energy state (Figure 1B). The optical resonances in SWCNTs is inherent to excitons (electron-hole pairs) (Wang et al., 2005; Avouris et al., 2008). The local dielectric environment and charge transfer can affect the fluorescence of SWCNTs (Valerie C. Moore et al., 2003; Strano et al., 2003; Chen et al., 1998).

Compared with organic fluorescence dyes, SWCNTs are more stable without blinking or photobleaching (Heller et al., 2005). The electronically unidirectional structure related van Hove is shown in Figure 1A. The E_ii_ is considered as the energy gap between valence and conduction bands. The valence bands absorb photon of energy E_22_ and emit lower energy of E_11_ within the near-infrared region (Figure 1C). The roll-up vector from (0, 0) to (n, m) of graphene sheet determine energy values of E_ii_. The Kataura plot shows the relationship between the band gap, diameter, and chirality of SWCNTs, which is a diagram introduced by Kataura and colleagues synthesized SWCNTs using laser vaporization method and further clarified the inter-relationship between the energy gap, diameter, and roll-up vector (Saito et al., 2017). The local environment will affect the SWCNTs physical and chemical properties because of the outmost carbon atoms (Cognet et al., 2007; Heller et al., 2009).

Surface modification of SWCNTs can decrease the cytotoxicity and improve water solubility. Multiple biologically important molecules and polymers can bind to SWCNTs via electrostatic interactions to obtain high-performance SWCNTs for biomedical application (Figure 1F). Biological compatibility molecular with hydrophilic groups are widely applied in modifying the SWCNTs to obtain better solubility (Figures 1D,E) (Dai, 2002; Richard et al., 2003; Qiao and Ke, 2006). Dang et al. (2011) reported the combination of SWCNTs and TiO_2_ to form core/shell nanocrystal composites to increase the electron collection efficiency that greatly improved the power conversion efficiency. In general, SWCNTs showed strong fluorescence and low photobleaching in NIR-II, achieving deep tissue penetration and high spatial resolution fluorescence imaging. However, the solubility, biocompatibility and quantum yield of SWCNTs are poor, which makes it impossible to be directly used in bioimaging before surface modification. Nano-engineering of SWCNTs offers both biological compatibility and fluorescence properties for biosensing and bioimaging.

### Rear Earth Nanoparticles

Nanoparticles doped with rare Earth elements have unique optical, electrical and magnetic properties (Ma et al., 2016; Fan and Zhang, 2019; Han et al., 2020). The rare-earth nanoparticles (RENPs) have the special optical characteristics of various emission wavelength, long lifetime non-photobleaching and large stokes shift (Ma et al., 2016; Li D. et al., 2020; Zhou et al., 2020). The strategy of doping and regulating the morphology endow the RENPs with excellent NIR optical properties (Liang et al., 2015; He et al., 2020).

**FIGURE 2:**
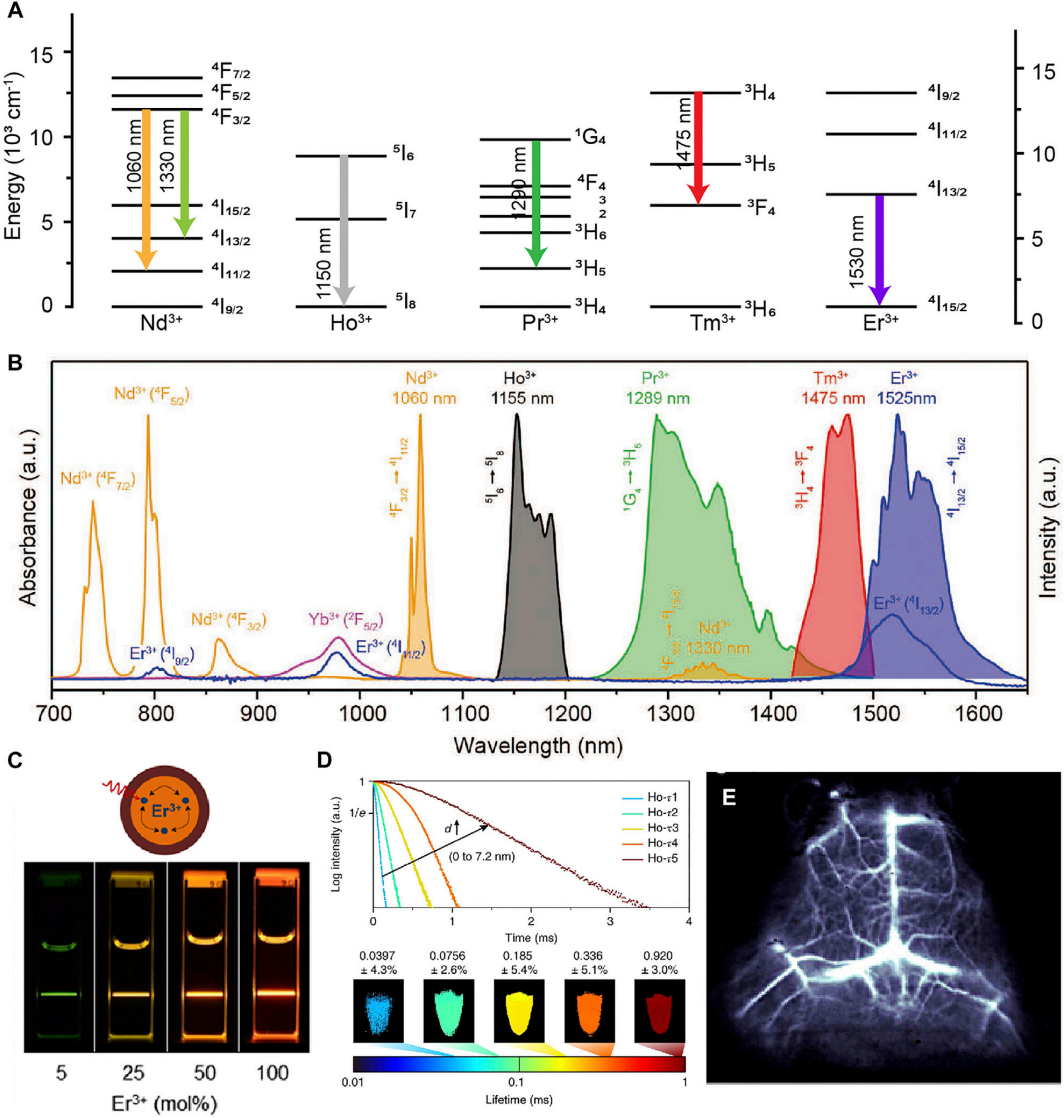
**(A)** Schematic energy level diagrams of trivalent lanthanide ions activators (Ln = Nd^3+^, Ho^3+^, Pr^3+^, Tm^3+^, Tm^3+^). **(B)** Intrinsic NIR emissions from Nd^3+^, Ho^3+^, Pr^3+^, Tm^3+^, Tm^3+^ (Fan and Zhang, 2019). Reproduced with permission from Fan and Zhang, (2019). **(C)** Schematic illustration of the NaErF_4_@NaLuF_4_ core/shell nanocrystals structural composition (Johnson et al., 2017). Reproduced with permission from Almutairi et al. (2017). **(D)** Luminescence decay curves of Ho-doped core-multi-shell nanocrystals and lifetime images measured at 1,155 nm (Fan et al., 2018). Reproduced with permission from Zhang et al. (2018). **(E)** Cerebral vascular image in 1,550 nm of β-NaYbF_4_:Ce,Er@NaYF_4_ nanoparticles (Zhong et al., 2017). Reproduced with permission from Dai et al. (2017).

With abundant f orbital electrons, Ln^3+^ ions can exhibit multi-wavelength emissions that are commonly used as emitting centers (Wen et al., 2018; Cao et al., 2019). Lanthanide-doped nanoparticles are composed of host matrix, sensitizer, migrator and activator. Host matrix is necessary, in which trivalent lanthanide ions are embedded. The host matrix with suitable lattice dimensions can provide a crystalline environment, which have the characteristics of low lattice phonon energy, negligible absorption and high stability. The sensitizer ion can harvest pump photons and subsequently promote the nearby accumulator ion to excited states. The migrator ion act as an energy bridge between sensitizer and activator. To obtain tunable emissions in the NIR spectral region (700–1700 nm), Ho^3+^, Pr^3+^, Nd^3+^, Tm^3+^ and Er^3+^ are usually used as the activators (Figures 2A,B; Fan and Zhang, 2019). Except the ETU process, the directly photon absorption of activators has emerged as an attractive way with high quantum yield (Figure 2).

Deleterious cross-relaxation, surface quenching effects and nonradiative relaxation induced by host matrix seriously quench the NIR luminescence. Liu group constructed a series of fluorescence nanomaterials with tunable upconversion emissions by introduced the core-shell structure to eliminate the deleterious cross-relaxation (Wang et al., 2011). This study open opportunities toward exploring a new class of luminescent materials (Chen et al., 2015; Labrador-Paez et al., 2018; Wang et al., 2014; Yan et al., 2019; Zhao et al., 2020). Concentration quenching once was a major barrier in designing materials. Dai group described the relationship between the doping concentration and surface quenching effects. They found that the inert outer shell could overcome surface quenching effects (Figure 2C; Johnson et al., 2017). Nanoparticles doped with appropriate lanthanide ions has proved to enhance NIR emission through energy trapping or suppress the up-conversion pathway (Chen et al., 2017; Zhong et al., 2017). Fluorescence lifetime as a new dimension become highly useful in multichannel bioimaging (Figure 2D), which are independent of both colour and intensity (Laviv et al., 2016; Fan et al., 2018; Zheng et al., 2018; Xie et al., 2020; Lukina et al., 2021; Luo et al., 2021). Zhang group designed NIR-II RENPs with tunable luminescence lifetimes for *in vivo* quantitative imaging (Fan et al., 2018). RENPs have attracted more and more attention due to their large Stokes shift, narrow and multi-peak emission spectra, negligible excitation-emission band overlap, and excellent photostability. However, the long residence time in the reticuloendothelial system and cannot be removed from organism. which increases the potential safety hazards and is a non-negligible obstacle for future biomedical applications.

### Quantum Dots

**FIGURE 3:**
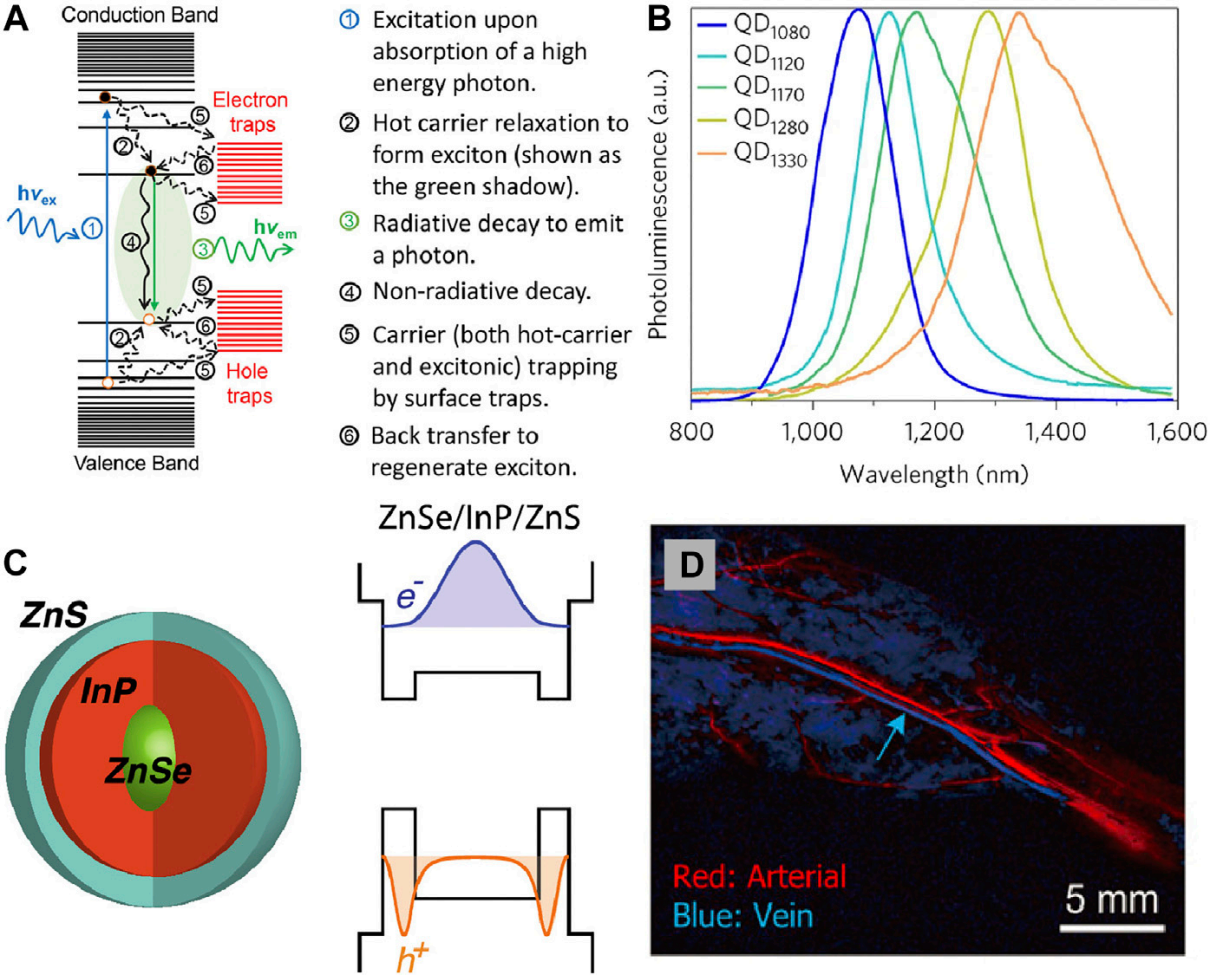
**(A)** Schematic of basic processes involving various excited states in a quantum dot upon photoexcitation (Pu et al., 2017). Reproduced with permission from Peng et al. (2017). **(B)** Spectra of different core/shell short-wave infrared quantum dots: QD_1080_ (InAs(CdSe)_1_(ZnSe)_3_); QD_1120_ (InAs(CdSe)_1.5_); QD_1170_ (InAs(CdSe)_3_); QD_1280_ (InAs(Cd_0.9_Zn_0.1_S)); QD_1330_ (InAs(CdSe)_6_) (Bruns et al., 2017). Reproduced with permission from Bruns et al. (2017). **(C)** NIR emission ZnSe/InP/ZnS core/shell/shell nanoparticles and their bandgap alignments with corresponding electron and hole wave functions (Saeboe et al., 2021). Reproduced with permission from Dennis et al. (2021). **(D)** PCA for differentiation of arterial and venous components (Zhang et al., 2018). Reproduced with permission from Zhang et al. (2018).

Quantum Dots (QDs) are colloidal semiconductor nanocrystals with few nanometres in size. With distinct electronic and optical properties benefiting from smaller radii and quantum confinement effects, QDs display a promising application prospect in biomedical systems as ideal probes for fluorescence imaging (Figure 3D).

The unique optical properties of QDs depend on the transition of an electron from the valence band to the conductance band. When their radii become smaller than the exciton Bohr radius, the excitations are squeezed that lead to the quantum confinement effect. In semiconductor nanocrystals there is a band-gap between the valence band and conductance band. It generally leads to the an electron excited to the conductance band, when a photon absorption in the valence band with appropriate energy to band-gap energy. This process produce quasi-particle called exciton consist of the negatively charged electron and the positively charged hole. When the excited electron resumes its ground state, it eliminates the exciton and release energy in the shape of a photon through radiative recombination (Figures 3A,B). In the quantum confinement system, the emission wavelength and band gap energy of QDs can be tuned by regulating their size, materials et al. (Hanson et al., 2017; Grimaldi et al., 2019; Santos et al., 2020; Sarkar et al., 2020; Yang et al., 2021). It is possible to achieve QDs with NIR light emission by selectively control the composition process, endowing QDs superior imaging capability (Hong et al., 2017; Cao et al., 2019; Sonego et al., 2019; Gil et al., 2021).

The hydrophobic and toxicity of the NIR QDs limits their *in vivo* application. Several alternatives have been developed such as toxic elements free or surface coating to endow the NIR QDs more suitable for *in vivo* applications. It was found that PEG, MPA, and SiO_2_ increased their circulation time (Akerman et al., 2002; Gussin et al., 2006). The emission wavelength of traditional binary QDs systems such as CdSe, CdTe, and PbS always in the visible region. In order to reduce biological toxicity, the heavy metal-free QDs are attracting more and more attention. QDs luminescent in biologically desired NIR region can be obtained by the strategies: 1) changing the size of QDs, 2) dopping different ions into the host matrix, 3) rational design of core-shell structure. Bawendi designed a series of copper indium selenide QDs by changing the stoichiometries that can exhibited fluorescence from red to near-infrared (NIR) (Allen and Bawendi, 2008). By using the toluene thermal and a hot-injection method, Deng et al. (2009) synthesised quaternary Cu_1.0_Ga_x_In_2-x_S_3.5_ and Cu_1.0_In_x_Tl_2-x_S_3.5_ QDs with NIR emission. Redshift of luminescence was observed when the doped ions content was increased, which is great importance for labeling and imaging studies. By introducing HgS interlayer at the core/shell CdSe@CdS QDs interface, the visible emitters can be converted into highly efficient NIR fluorophores by Klimov (Sayevich et al., 2021). Dennis also reported the similar ZnSe@InP@ZnS core/shell/shell heterostructure, in which tunable emission ranging from visible to NIR wavelengths can be obtained by changing the InP interlayer thickness (Figure 3C; Saeboe et al., 2021). Through well-designed core-shell structure, doping different ions, and surface modification the NIR emissions QDs with biocompatible properties can be achieved. As a semiconductor fluorescent nanoprobe, QDs has good optical and chemical stability, narrow and tunable emission wavelength, high two-photon absorption cross section, and polychromatic fluorescence imaging. Although, the current synthesis approach and surface functional modification methods have reduced the biological toxicity of QDs, but its safety is still controversial, which may become a potential risk factor for clinical application.

### Metal Nanomaterials

In material science, metal nanomaterials are another type of inorganic nanomaterials that are promising for NIR bioimaging. Meanwhile, binary or ternary metal nanomaterials can greatly expand the properties of metals. Researchers in material science have discovered that some metal nanomaterials not only demonstrate outstanding catalytic properties, but also have excellent optical properties (Wang S. et al., 2020; Li H. et al., 2020; Shu et al., 2020; Sun et al., 2021; Zhou et al., 2021).

**FIGURE 4:**
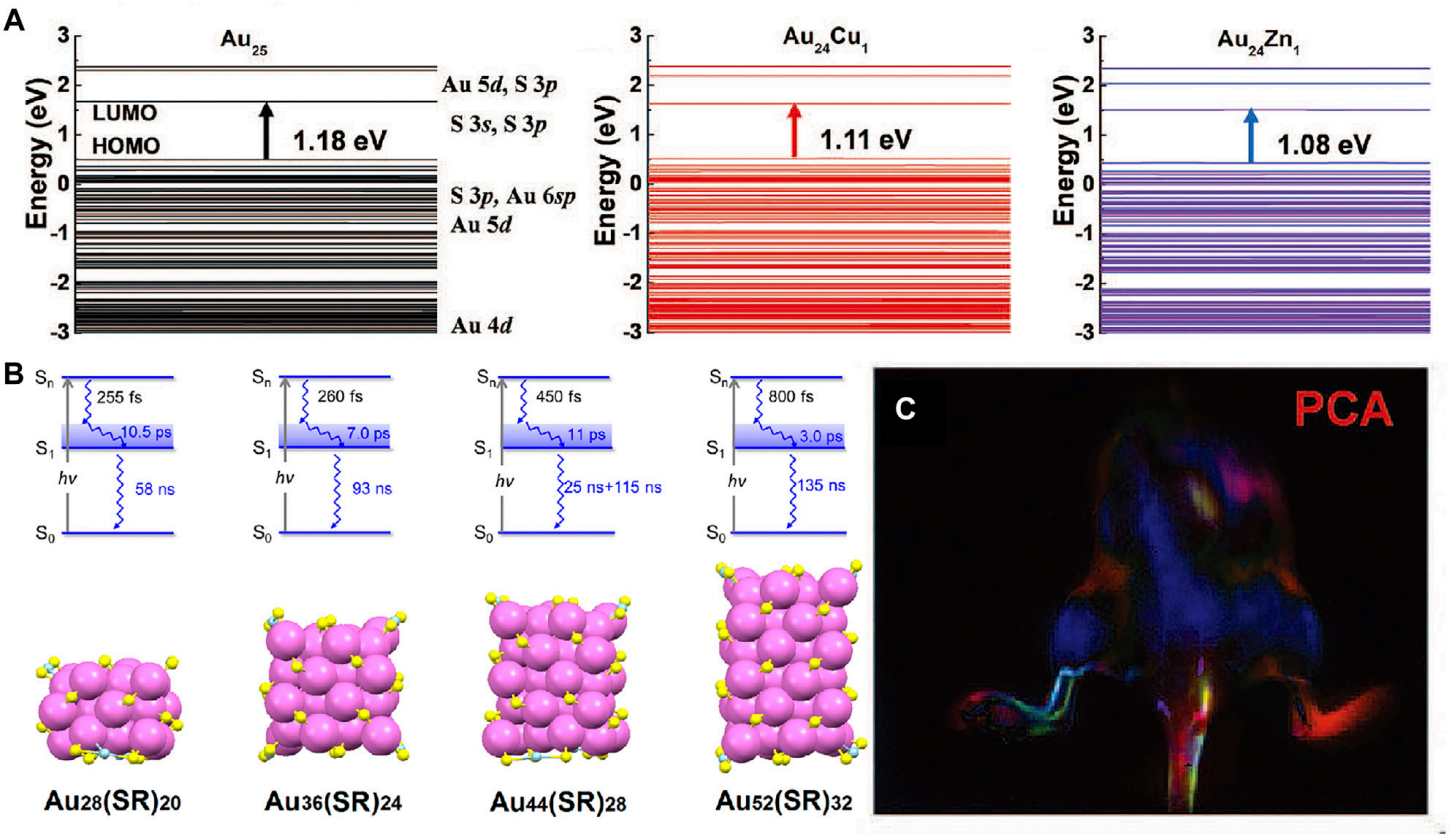
**(A)** Schematic of the energy level of Cu and Zn doped Au_25_ clusters by density functional theory (Liu et al., 2019). Reproduced with permission from Zhang et al. (2019). **(B)** Relaxation model and the corresponding Au_m_ (SR)_n_ nanocluster crystal structures (Zhou et al., 2017). Reproduced with permission from Jin et al. (2017). **(C)** Dynamic NIR-II fluorescence imaging of lymphatic metastasis with Au_25_ clusters and principal component analysis images (Liu et al., 2019). Reproduced with permission from Zhang et al. (2019).

Different from traditional noble metal nanocrystals with unique optical properties dominated by surface plasmon resonance, the metal nanoclusters have strong quantum confinement effect with discrete energy band. Affected by quantum size effect, the absorption spectra is associated with both the metal core and surface states. Under both near HOMO-LUMO gap and higher energy excitations, one can observe core-to-shell charge transfer for the nanoclusters (Figure 4A). As the size of the metal core increases, the energy gap between HOMO and LUMO decreases slowly corresponding to absorption spectrum. Similar to the trend in absorption spectra, the emission wavelength of the metal nanoclusters show red-shifted evolution. (Yao et al., 2017; Zhang et al., 2019; Wu et al., 2020; Li et al., 2021; Yu et al., 2021).

Novel gold nanoclusters have been developed to emit strong fluorescence in NIR-II region as well as quick renal clearance (Liu et al., 2019; Li et al., 2021). Jin group reported the femtonanosecond excited state dynamics of a periodic series of face-centered cubic gold nanoclusters with NIR emission (Figure 4B), which will improve optical energy harvesting and extend photocatalytic applications (Zhou et al., 2017). Doping is another effective strategy for improve the NIR property of the metal nanomaterials (Andolina et al., 2013; Marbella et al., 2014). Zhang group presented a unique cage-like Au_25_ cluster that can emit fluorescence in 650–1,400 nm region by the charge transfer between outside glutathione and inner Au core (Figure 4C). They found that the corresponding fluorescence QY is remarkably improved by Cu, Zn, Ag, and Er doping (Liu et al., 2019). In recent years, a series of near-infrared fluorescent metal nanoprobes with excellent NIR performance have been developed. Metal nanomaterials show higher quantum yield and lower photobleaching. It is often used for liver, kidney, brain and lung imaging. However, these materials tend to stay and accumulate in the liver and spleen, which are not easily excreted by the body.

## Biological Application

With the rapid development of inorganic NIR fluorophores, more and more researchers applied the NIR fluorophores in bioimaging and biosensing applications. In this part, we summarized some representative works on inorganic NIR fluorophores biological applications, including precise diagnosis, lymphatic or vessel system imaging, image-guided surgery and therapy.

### Precise Diagnosis

**FIGURE 5:**
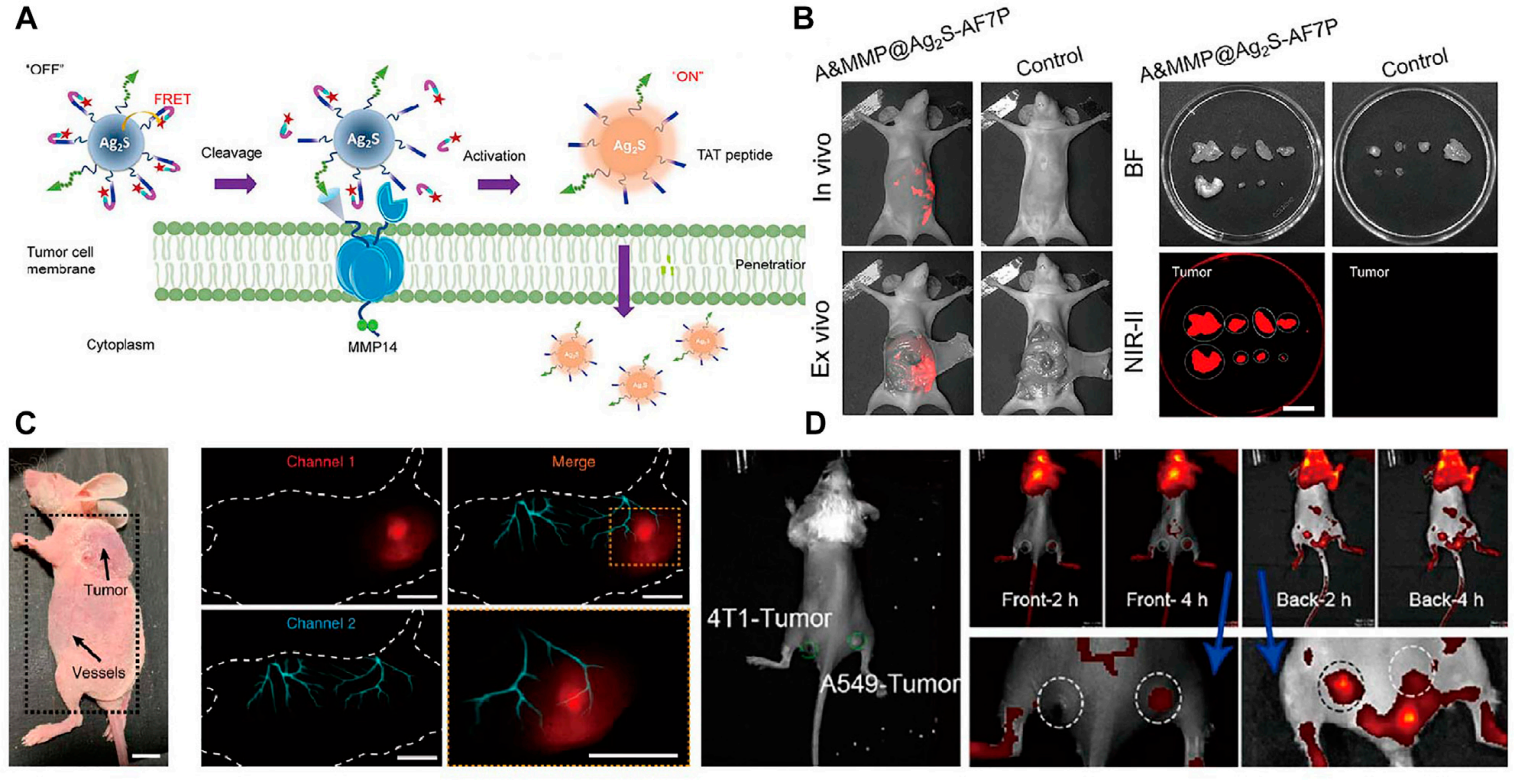
Representative reporters of tumor diagnosis using NIR fluorescence. **(A)** Schematic illustration of A&MMP@Ag_2_S-AF7P for NB detection. **(B)** A&MMP@Ag_2_S-AF7P NIR-II fluorescent and bright field image of excised tumor nodules for pre-operative and postoperative stage of peritoneal tumor (Zhan et al., 2021). Reproduced with permission from Wang et al. (2021). **(C)** Dual-channel NIR bioluminescence imaging of CT-26 tumor tissue (upper) and vessels (lower). (Lu et al., 2020). Reproduced with permission from Zhang et al. (2020). **(D)** *In vivo* NIR imaging of the mouse inoculated with 4T1 and A549 tumor cells (Lv et al., 2021). Reproduced with permission from Tian et al. (2021).

Scientists have paid more attention to the tumor visualization, which will help to improve deeper understanding of the mechanisms of tumor metastasis, accurate diagnosis and treatment. Presently, NIR imaging has been widely applied for diagnosis of tumor in early stages with high resolution (Wang et al., 2018a). Construction of tumor microenvironment responsive probe, especially toward specific enzyme of solid tumors, is a significant task. Precise intraoperative histopathological analysis are complicated, which greatly delay the intraoperative decision. Zhan et al. (2021) successfully developed matrix metalloproteinase 14 (MMP14) activated (A&MMP@Ag_2_S-AF7P) for accurate NIR imaging diagnosis of neuroblastoma (NB). In this nanoprobe, the affinity peptide can help to recognize and target the NB cells by overexpressed matrix metalloproteinase 14. Afterwards, internalization of the nanoprobes was occurred through specific enzymatic reaction of affinity peptide and matrix metalloproteinase 14. NIR-II fluorescence emission of Ag_2_S QDs was recovered through cut off the FRET process between Ag_2_S quantum dots and NIR absorber (Figures 5A,B). Well-defined tumor margins and instant illumination of the lesion make it an ideal diagnostic nanoprobes for cancer surgical. For fluorescence imaging in biological organs and tissues, autofluorescence, scattering and absorption coefficient are still primary obstacles. Lu et al. (2020) designed bioluminescence probes with emission at NIR-II window by introducing BRET and two-step FRET process. The strategy highly improved imaging resolution of tumors, metastases, vessels and lymphatics. The high solution multiplexed imaging using NIR-II bioluminescence probes is shown in Figure 5C. RENPs have been widely studied in the biomedical field. In 2018, Xu et al. (2019) have summarized the recent advances of lanthanide-doped nanoconstructs in biomedical imaging. Fluorescence imaging probes combined the antibody of the searched hub genes with NaYF_4_:Yb,Er,Eu@NaYF_4_:Nd NPs with orthogonal emission properties are designed for precise diagnosis of lung adenocarcinoma (Lv et al., 2021. Figure 5D). Tao et al. (2017) reported a NIR imaging nanoprobes consist of lanthanide ion doped nanocrystals coating with PEO-b-PCL polymers and doped with carbocyanine dye that facilitates precise detection of smaller and earlier tumor deposits.

There are other reports for diseases diagnosis and neuronal activity tracking using NIR fluorescence imaging. Shemetov et al. (2021) efficiently detected changes in visually evoked neuronal activity in the primary visual cortex of head-fixed awake mice though a near-infrared genetically encoded calcium indicator. Fast and accurate diagnosis is crucial for treatment in clinical medicine. Fan et al. (2017) aimed at developing a strategy for fast and cost-effective diagnosis of kidney disease using NIR spectra of human serum. Chen et al. (2020b) introduced a second near infrared fluorescence imaging strategy based on lead sulfide quantum dots dynamically monitoring bacterial infection in a real-time manner. A gold nanostar based near-infrared fluorescence imaging probe is designed by Hua et al. (2021) for labeling and precise tracking of the stem cells, as a potential approach for the precise diagnosis and treatment of myocardial infarction. Keloid scars always accompany with an abnormal high reactive oxygen and nitrogen species level.

### Vessel and Lymphatic Imaging

**FIGURE 6:**
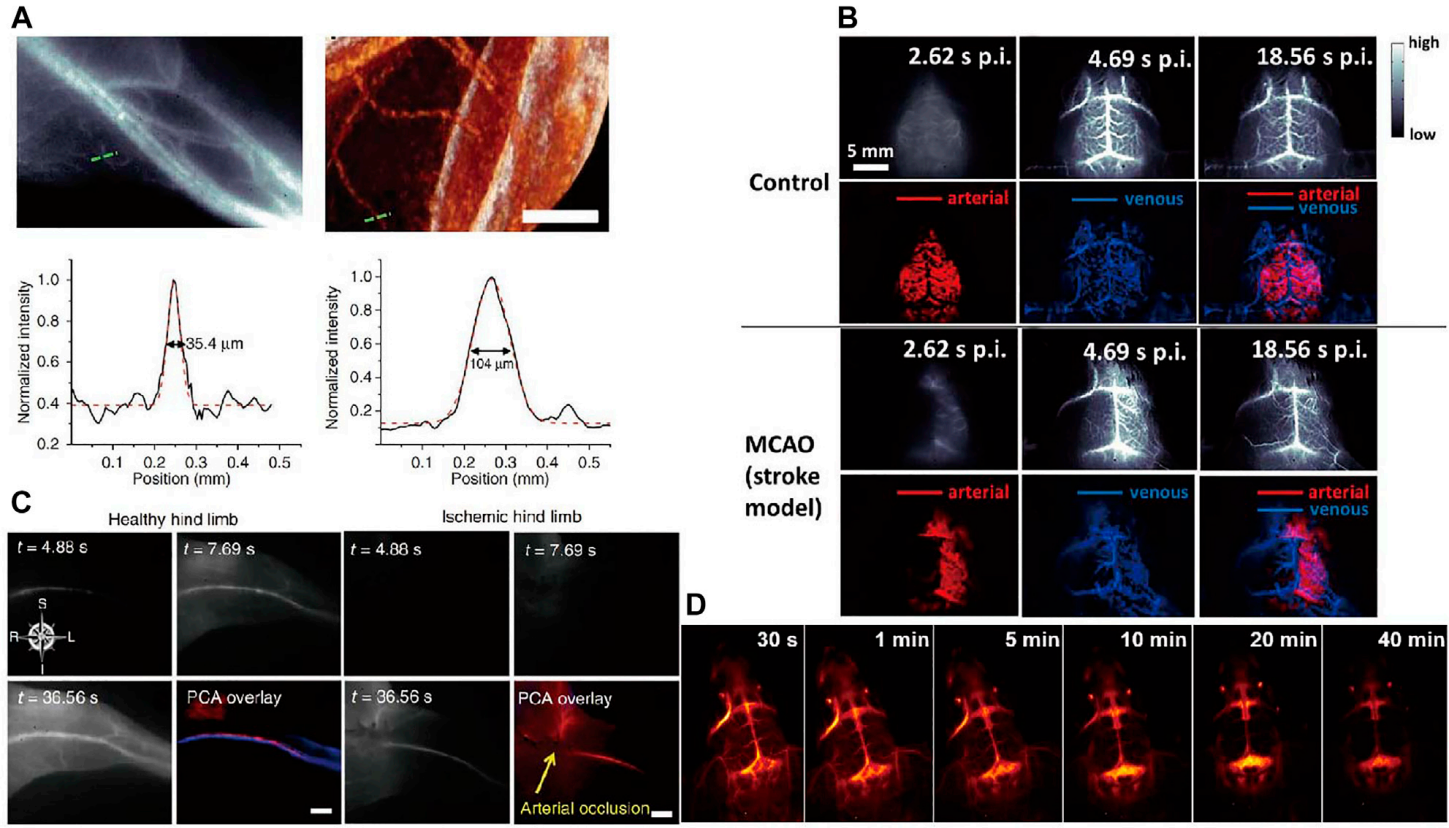
Vessel imaging. **(A)** Comparison of NIR-II fluorescence and micro-CT imaging of hind limb blood vessels (Hong et al., 2012). Reproduced with permission from Dai et al. (2012). **(B)** Dynamic NIR fluorescence imaging of mouse cerebral vasculature (Hong et al., 2014). Reproduced with permission from Dai. (2014). **(C)** Principal component analysis (PCA) of femoral artery and vein in healthy and ischemic mice by NIR-II imaging. (Hong et al., 2012). Reproduced with permission from Dai et al. (2012). **(D)** Real-time imaging of brain vessels using PAA-modified β-NaErF_4_@NaYF_4_ NPs through intravenous injection via the tail vein (Li et al., 2020c). Reproduced with permission from Chen et al. (2020a).

Vessel imaging is closely connected with life health that can provides anatomic and hemodynamic information. However, imaging techniques such as CT and MRI for assessing vasculature and hemodynamics are limited by the long scanning and postprocessing times (Saba et al., 2010; Greco et al., 2012). Due to high temporal and spatial resolution, *in vivo* NIR fluorescence imaging has inherent advantages over tomographic imaging (Adams et al., 2002). The resolution limits between NIR-II and micro-CT imaging of hind limb vasculature as well as the cross-sectional signal intensity are displayed in Figure 6A. In the distal hind limb of the mouse, the NIR-II image showed larger numbers of small blood vessels compared to the micro-CT image at the same location. The smallest vessel extracted by NIR-II had a diameter of only 35.4 µm, whereas micro-CT could not identify any vessel smaller than ∼100 µm in diameter. The temporal as well as spatial resolution far exceeds scanning microscopic imaging techniques. Hong et al. (2014) reported NIR fluorescence imaging of mouse cerebral vasculature using single-walled carbon nanotubes in an acute stroke model in mice, which provided real-time assessment of blood flow abnormalities. Noninvasive, high-resolution NIR fluorescence images of cerebral vasculature are shown in Figure 6B.

There are other representative blood vessels fluorescence imaging works. Highly bright fluorescence probe with long-wavelength absorption and aggregation-induced NIR emission is designed by Wang et al. (2019) for mouse brain and tumor vasculatures imaging. Hong et al. (2012) reported brightly, biocompatible NIR imaging probes (SWCNTs) for vascular imaging in mouse hind limb with high spatial resolution of ∼30 µm. Principal component analysis (PCA) can distinguish arteries from veins to obtain high contrast dynamic imaging by using NIR fluorescence imaging. NIR fluorescence imaging of femoral artery and vein of hind limb in healthy and ischemic mice is shown in Figure 6C. Ma et al. (2018) reported the NIR-II imaging of hind limb microvasculature and blood perfusion using a peripheral arterial disease mice model. They used NIR fluorescence contrast agent PbS/CdS to quantify vascular hemodynamics and vascular structures, which can provide real-time monitoring of tissue infusion recovery and neovascularization in avascular limb. High-magnification imaging of vascular regeneration and quantification graph in the ischemic hind limb is shown in Figure 6D.

**FIGURE 7:**
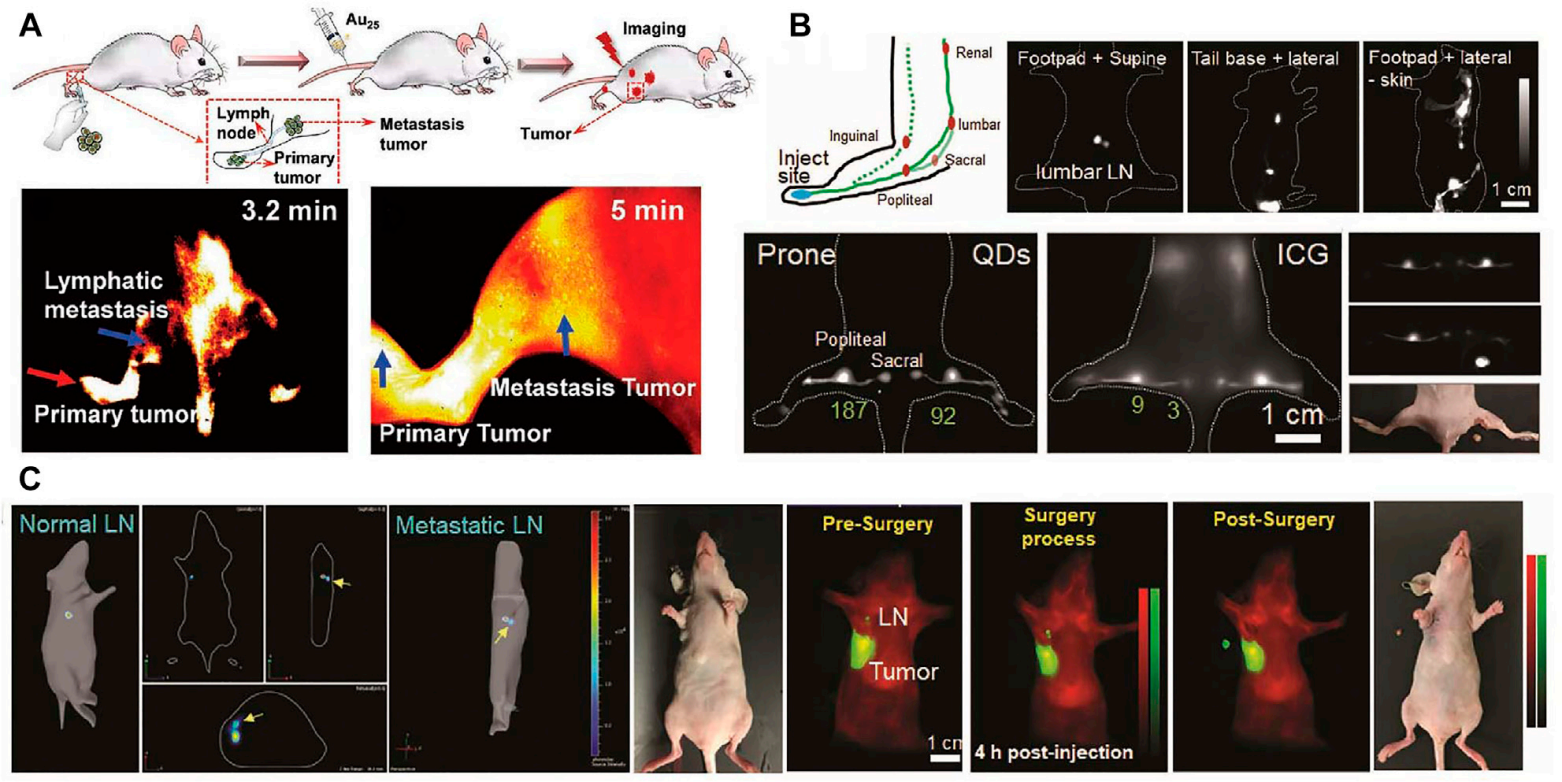
Lymphatic imaging. **(A)** Schematic graphic of tumor metastasis, dynamic primary tumor and lymphatic metastasis imaging (Liu et al., 2019). Reproduced with permission from Zhang et al. (2019). **(B)** Schematic illustration of lymphatic system in the hindlimb, lumbar, renal lymph nodes and the advantages of QDs in popliteal and sacral lymph nodes imaging compared with ICG; NIR imaging guided surgery of popliteal lymph node. **(C)** The dual NIR imaging guided sentinel lymph node surgery in orthotopic 4T1 breast cancer model (Tian et al., 2020). Reproduced with permission from Chen et al. (2020b).

Lymphatic system provides an accessory return route to the blood consist of lymphatic vessels, lymph nodes, and other lymphatic organs that plays an important role for the maintenance and immunocompetence fluid homeostasis. Cancer cell migration from primary tumor to sentinel lymph nodes is a remarkable prognostic indicator for cancer evolution. Hence, the accurate diagnosis of lymph node metastasis is necessary for further treatment strategies (Yaniv et al., 2006; Trevaskis et al., 2015). Liu et al. (2019) developed a gold cluster with efficient NIR fluorescence. In that work, High-resolution imaging of nanomaterials allows for the identification of the lymphatic metastasis and primary tumor. Schematic graphic of tumor metastasis, dynamic primary tumor and lymphatic metastasis imaging is shown in Figure 7A. Kim et al. (2004) reported CdTe@CdSe core/shell type II quantum dots for sentinel lymph nodes biomedical imaging with only excitation fluence rates of 5 mW/cm^2^. The outstanding *in vivo* fluorescence imaging results presented in that work demonstrated the advantages of NIR quantum dots for sentinel lymph node imaging.

NIR photoacoustic imaging is an attractive strategy for accurate diagnosis of sentinel lymph node (Pan et al., 2009). Exogenous NIR contrast probes are needed for photoacoustic imaging of the lymph nodes. Pan et al. (2010) have designed NIR gold nanobeacons for precise detection of sentinel lymph node. In a recent work, Kang et al. (2017) reported the integrated NIR/gamma/visible tri-modal fusion imaging system for sentinel lymph node imaging in C57/B6 mouse. Real-time visualizing regional lymphatic metastasis and operating imaging-guided proximal lymph node resection surgery still have many challenges. Tian et al. (2020) reported a multiplexed-near-infrared imaging nanomaterial of non-overlapping NIR agent with significantly reduced scattering and negligible autofluorescence. In this study, PbS@CdS core/shell QDs with dense PEG coating are used to realize tumor invaded sentinel lymph node dual-NIR imaging. As shown in Figure 7B, compared to clinically applied ICG organic dyes, the QDs show favorable photostability and high contrast. The NIR fluorescence imaging guided sentinel lymph node removal surgery is displayed in Figure 7C.

### Imaging-Guided Surgery

**FIGURE 8:**
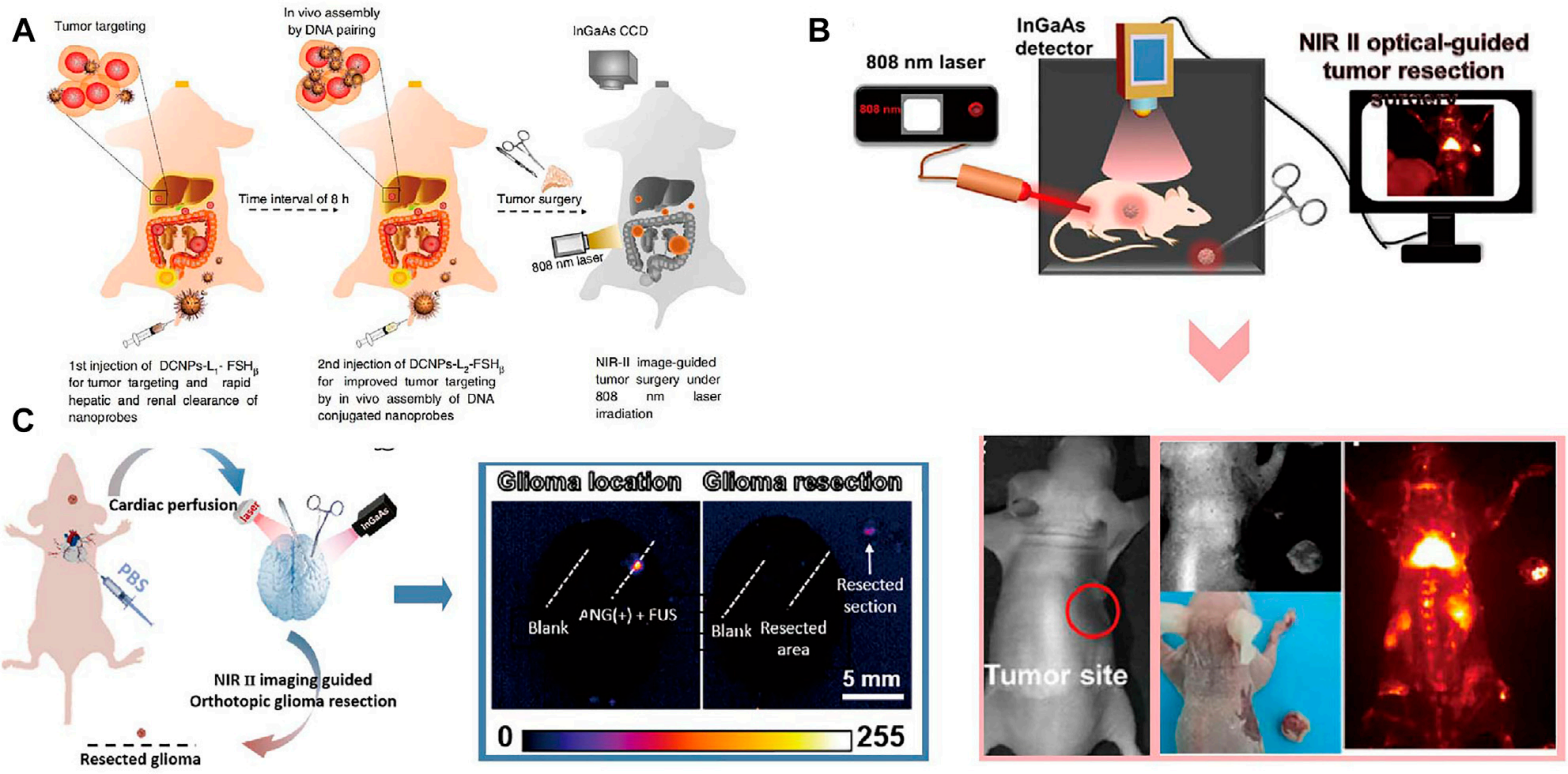
Imaging-guided Surgery. **(A)** Schematic illustration of NIR-II optical imaging-guided surgery of ovarian metastasis (Wang et al., 2018a). Reproduced with permission from Zhang et al. (2018). **(B)** The NIR-II bioimaging guided tumor resection of the colorectal tumor-bearing mouse (Li et al., 2020b). Reproduced with permission from Zeng et al. (2020). **(C)** Schematic illustration of NIR- II fluorescence imaging guided resection of orthotopic glioma (Ren et al., 2020). Reproduced with permission from Li et al. (2020c).

NIR imaging as a powerful tool for early detection of malignant lesions in clinical has been used for imaging-guided surgery in cancer diagnosis and treatment (van Dam et al., 2011). Recently, Hu et al. (2020) described an NIR imaging instrument for the fluorescence-guided surgical resection of primary and metastatic liver tumor. Tumor metastasis is a significant cause of treatment failure for primary tumor. Lanthanide based nanomaterials with high efficient downshifting near-infrared emission has emerged as a promising agent for imaging-guided resection surgery of tumor. Recently, DNA has attracted increasingly interest as a functional biomolecule to construct multimodal nanomaterials. Liu et al. introduced recent progress of DNA-functionalized upconversion materials, providing a comprehensive overview of applications in biomedicine (Liu et al., 2018). Wang et al. (2018b) designed a down-shifting NIR-II nano-probes grafted with specific DNA sequence that can turned into larger assembled superstructures to improve the image-guided surgery for metastatic ovarian cancer (Figure 8A). Larger assembled superstructures improved tumor-to-normal tissue ratio that facilitate the abdominal ovarian metastases surgical delineation. Searching for suitable NIR fluorescence agents play an important role in imaging-guided surgery. Li et al. (2020d) reported the Nd-sensitized lanthanide-based nanoprobe with up to 11.0 times improved NIR-II emission intensity for imaging-guided surgical resection of tumor. Schematic illustration and colorectal tumor-bearing mouse of the NIR-II optical imaging-guided tumor resection are shown in Figure 8B. Limited by deep tissue penetration and high efficiency of crossing the blood-brain-barrier, the development of imaging-guided surgery of brain tumors was significantly constrained. Ren et al. (2020) reported a Er-based DCNPs with strong NIR-II fluorescence in fluorescence-guided surgery of orthotopic glioma through intact skull and scalp. The high tumor-to-background ratio in NIR-II fluorescence imaging of small orthotopic glioma ensure the precise resection of tumor (Figure 8C). Overall, the emergence of NIR imaging shows great promise for improving the specificity, efficiency, and safety of imaging-guided surgery.

### Disease Theranostics

**FIGURE 9:**
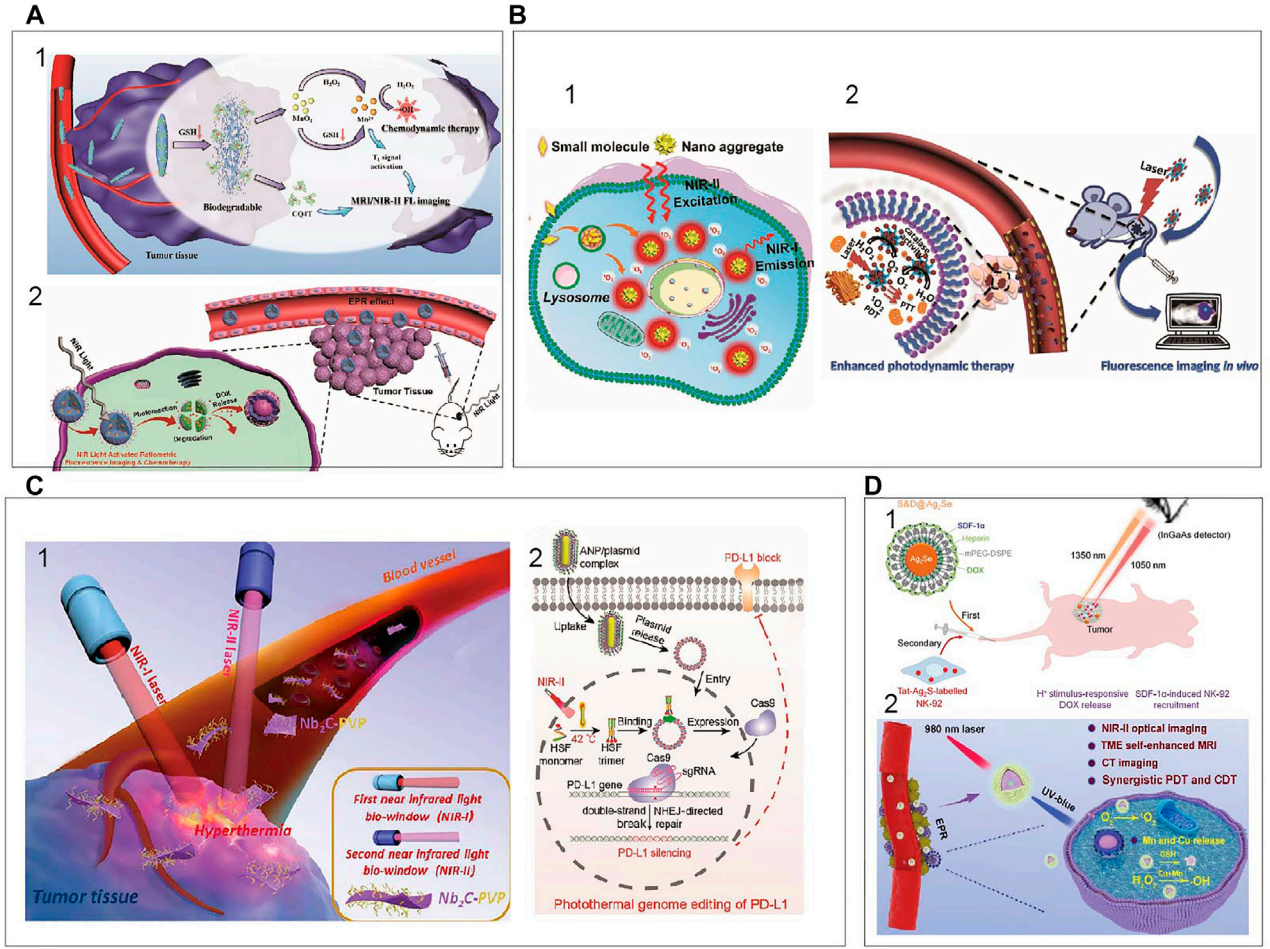
An illustrated summary of significant CDT, PDT, PTT **(A) (i)** Schematic illustration of FMSN-MnO_2_-BCQ for dual-mode imaging and self-reinforcing CDT (Zheng et al., 2021). Reproduced with permission from Zheng et al. (2021). **(ii)** NIR-light-regulated fluorescence imaging and chemotherapy (Chen et al., 2020a). Reproduced with permission from Chen et al. (2020b). **(B) (i)** NIR-II light activated two-photon photodynamic cancer ablation (Wang et al., 2020a). Reproduced with permission from Liu et al. (2021). **(ii)** Application of Pd@Pt-PEG-Ce6 (Wei et al., 2018). Reproduced with permission from Zheng et al. (2018). **(C) (i)** 2D biodegradable Nb_2_C for photothermal tumor ablation in NIR windows (Lin et al., 2017). Reproduced with permission from Shi et al. (2017). **(ii)** Photothermal activation for PD-L1 genome editing in tumor (Tang et al., 2021). Reproduced with permission from Ping et al. (2021). **(D) (i)** NIR fluorescence imaging to program the chemotherapy and immunotherapy (Hao et al., 2018). Reproduced with permission from Wang et al. (2018a). **(ii)** PEG/LDNPs@CMSNs for TME and NIR laser co-enabled PDT/CDT (Xu et al., 2020). Reproduced with permission from Lin et al. (2020).

Recently, large amount of NIR based theranostics strategies have been developed. NIR fluorescence based therapy including chemodynamic therapy (CDT), photothermal therapy (PTT) and photodynamic therapy (PDT) is gradually emerged for cancer treatment. How to take advantage of the tumor microenvironments characteristics to better image diseases and combine efficient therapeutics is still a challenge. To solve this problem, Zheng et al. (2021) have developed the FMSN-MnO_2_-BCQ biodegradable nanoplatforms, with TME-activated tumor-deep delivery performance for accurate imaging and self-reinforcing CDT (Figure 9Ai **)**. In 2020, Chen et al. (2020c) have successfully prepared a NIR-light triggered ratio metric fluorescent nanoplatform composed of UCNPs, DOX, and photosensitive block copolymer PEG-b-P(NBA-co-NBANA) (Figure 9Aii **)**. This research provides a new strategy to achieve high spatial-temporal-controlled biological imaging and chemotherapy. An illustrated summary of significant CDT is shown in Figure 9A. NIR light excitable photosensitizers are highly desirable for photodynamic therapy with deep penetration. An AIE PS (TQ-BTPE) with long-wavelength absorption and high ^1^O_2_ generation efficiency was designed for NIR-II light activated two-photon photodynamic therapy (Wang et al., 2020b). Schematic illustration of NIR-II light activated two-photon photodynamic cancer cell ablation is shown in Figure 9Bi. Upconversion nanoparticles (UCNPs) that can be excited by NIR light is an interesting topic in the field of PDT (Lee et al., 2020). UCNPs with a core-shell structure (NaYF_4_:Yb,Er,Nd@NaYF_4_:Yb,Nd) were synthesized to increase the upconversion emission efficiency. Dual-color emitting Er-doped UCNPs and dual photosensitizers were used for enhanced PDT. Inherent hypoxic nature of most solid tumors can heavily restrict the efficiency of PDT. A new nanoplatform based on Pd@Pt-PEG-Ce6 for enhanced photodynamic therapy by overcoming tumor hypoxia microenvironment is designed (Wei et al., 2018). Application of Pd@Pt-PEG-Ce6 is shown in Figure 9Bii. NIR light-mediated photothermal therapy has emerged as a powerful approach for cancer treatment. A series of nanomaterials absorbing NIR light to generate heat have been developed (Li et al., 2019). A novel kind of 2D niobium carbide (Nb_2_C), MXene, with highly efficient *in vivo* photothermal ablation of mouse tumor in both NIRI and NIR-II windows is explored (Lin et al., 2017). Schematic illustration of 2D biodegradable Nb_2_C (modified with PVP) for *in vivo* photothermal tumor ablation in NIR-I and NIR-II windows is shown in Figure 9Ci. This year, a novel photothermal genome-editing strategy is described to improve immune checkpoint blockade therapy. This strategy relies on a gold nanorod that not only serves as a carrier but also harvests NIR-II light and converts into mild hyperthermia to induce both immunogenic cell death (Tang et al., 2021). Illustration of photothermal activation for PD-L1 genome editing in tumor cells is displayed in Figure 9Cii.

As a paradigm-shifting treatment modality, immunomodulatory therapies have recently shown striking clinical efficacy in fighting the tumors (Jiang et al., 2021). Despite with promising clinical results, cancer immunotherapy is accompanied with severe side effects. Thus, it’s urgently needed to develop the smarter systems to regulate immune responses with superior spatiotemporal precision and enhanced safety. Chu et al. (2019) reported an activatable engineered immunodevice that enables remote control over the antitumor immunity *in vitro* and *in vivo* with near-infrared light. Combined chemotherapy and immunotherapy have demonstrated broad prospects in cancer treatment. A combinational administration chemotherapy and immunotherapy is programed to enhance the therapeutical effects (Hao et al., 2018). In that work, Ag_2_Se QDs loaded with chemodrug doxorubicin (DOX) and SDF-1α are first administrated to deliver the SDF-1α and DOX to the tumor site. After their arrival, natural killer (NK)-92 cells labeled with Ag_2_S QDs are intravenously injected so that the cells are recruited to the tumor by the chemotaxis of SDF-1α, which is visualized by Ag_2_S QDs NIR fluorescence. Schematic illustration of multiplexed QDs NIR imaging strategy to obtain the optimal synergistic therapeutical effects is displayed in Figure 9Di. CDT and PDT hold great promise for conquering malignant cancer. However, these methods are restricted by the overexpressed glutathione and hypoxia in the tumor microenvironment. Xu et al. (2020) develop biodegradable copper/manganese silicate nanospheres (CMSNs) coated lanthanide-doped nanoparticles (LDNPs) for NIR-II/MR/CT trimodal imaging-guided PDT/CDT synergistic therapy. As is shown in Figure 9Dii, the CMSNs can significantly relieve hypoxic through decomposing H_2_O_2_ to generate O_2_ which can react with the sample to produce ^1^O_2_ (PDT) and the GSH-triggered degradation of CMSNs results in the release of Mn^2+^ and Cu^+^ ions for ⋅OH generation (CDT). In 2021, an optimized AuND-based nanotheranostic platform was demonstrated for its capacity in cancer treatment with synergistic therapies of PTT and PDT in conjunction with the integrated different fluorescence imaging (Sun et al., 2021). It can be seen that the development of the bioapplications and the improvement of the NIR inorganic nanomaterials would be extended.

## Conclusion and Future Perspectives

In this review, we outline the inorganic NIR fluorophores that have evolved over the past decades. Numerous inorganic NIR nanomaterials including SWNCPs, lanthanide-based nanoparticles, semiconducting quantum dots and metal-based nanomaterials including the emission mechanism and biomedical applications have been discovered and reported. Many factors from the probes and imaging instruments that influence the high quality NIR-imaging of *in vivo* tissues and organs. To improve the NIR-imaging quality, the main research should be focus on the high quality optical probes and new imaging instrumentation. High-performance fluorophores are still limited by the absorption coefficients and fluorescence quantum yield. Further research on the fluorescence inorganic probes with highly quantum yield, suitable size, spectral properties (including the wavelength and fluorescence lifetime) and biological compatibility is important. Meanwhile, the development of new imaging instrumentation will be helpful to obtain deeper penetration depth, higher temporal and spatial resolution image. To efficiently deliver the probes *in vivo* effective modification strategies (ligand exchange, coating with phospholipid or polymer, modification with cell membrane or biomacromolecule, functional with targeting molecule et al.) should be taken and improved on the inorganic fluorescence nanoprobes. Effective modification of NIR nanomaterial should be taken to increase the biocompatibility and metabolic capacity. To expand the applications of inorganic NIR fluorophores in precise diagnosis and therapy, some challenges have to be overcome. Future efforts should be made to develop new fluorescence probes with multi-functions (diagnosis and treatment) and stimuli-responsive properties for precise theranostics. We looking forward to next era of multi-mode or multi-dimensions inorganic NIR fluorophores applied in bioimaging and biosensing. New imaging strategies such as bioluminescence or chemiluminescence imaging in the NIR-II spectral window without external excitation can better improve the signal-to-noise ratio. We aim to call on the research community to strengthen cooperation with scientists among diverse research fields, such as chemistry, biomedicine, and microscopy. Further investigations on both the more advanced cameras with higher sensitivity and broader NIR spectral ranges and new NIR inorganic nanomaterials are needed to be explored for precise diagnosis and therapy.
